# The Evolutionary Significance of Leaf Nodulation: Evidence from *Ardisia* and Its Relatives (Primulaceae: Myrsinoideae)

**DOI:** 10.3390/biology15171451

**Published:** 2026-08-24

**Authors:** Dan Wei, Tong-Jian Liu, Xiao-Kai Yan, Xin-Feng Wang, Ge-Han Huang, Yuan Xu, Xing Wu, Xue-Jun Ge, Gang Hao, Hai-Fei Yan

**Affiliations:** 1 College of Life Sciences, South China Agricultural University, Guangzhou 510642, China; 2 State Key Laboratory of Plant Diversity and Specialty Crops, South China Botanical Garden, Chinese Academy of Sciences, Guangzhou 510650, China; liutongjian@scbg.ac.cn (T.-J.L.);; 3 South China National Botanical Garden, Guangzhou 510650, China; 4 Guangdong Nanyue Ecological Technology Co., Ltd., Guangzhou 510500, China; 5 Suzhou Customs, Suzhou 215028, China; rphgh@126.com

**Keywords:** phyllosphere microbiome, leaf nodule, symbiosis, diversification rate

## Abstract

Interactions between plants and microorganisms have long been a central topic in biological research. Bacterial symbiosis on leaf surfaces represents a distinctive and mutually beneficial system within the phyllosphere microbiome. Leaf nodules are the visible manifestation of the symbiosis and confer ecological advantages to host plants by enhancing host resistance against pathogens and herbivores. It has been hypothesized that these advantages promote higher diversification rates in host lineages, but this remains uncertain. *Ardisia* subg. *Crispardisia* and its close relatives (*Amblyanthopsis* and *Amblyanthus*) within Primulaceae are typical plant groups with leaf nodule symbiosis, making them an ideal system for testing this hypothesis. In this study, we conducted extensive sampling of “Ardisioids” (*Ardisia* and its allies) and reconstructed their phylogenetic relationships and evolutionary history using plastid genomes and nuclear datasets (i.e., nuclear ribosomal DNA (nrDNA) and genome-wide single nucleotide polymorphisms (SNPs)). We clarified the phylogenetic positions of several “Ardisioids” genera (e.g., *Sadiria*, *Tapeinosperma*, *Amblyanthus*, and *Amblyanthopsis*) and multiple subgenera within *Ardisia*. We further detected a rapid radiation during the middle Miocene in *Ardisia* and its allies. Notably, we found that the leaf-nodulated clade appears to have originated during this period, approximately 11–8 Ma. BAMM (Bayesian Analysis of Macroevolutionary Mixtures) analyses revealed elevated diversification rates in leaf-nodulated lineages, while HiSSE (Hidden State Speciation and Extinction) analyses indicated that leaf nodule symbiosis might have increased speciation rates without significantly affecting extinction rates. These results provide strong evidence that leaf nodule symbiosis, together with other abiotic and biotic factors, represents a key evolutionary innovation that has promoted diversification in *Ardisia* and its close relatives.

## Simple Summary

Leaf nodule symbiosis, a unique bacterial association occurring in the phyllosphere microbiome, has been shown to influence host growth, immune responses, and secondary metabolism, suggesting its potential role in promoting evolutionary radiations. Nevertheless, direct macroevolutionary evidence linking this symbiosis to lineage diversification has remained scarce. In this study, we used *Ardisia*, a lineage characterized by typical leaf nodulation, to test this hypothesis. We reconstructed a robust phylogenetic framework based on plastid genomes, nrDNA, and genome-wide single nucleotide polymorphism (SNP) datasets. We resolved several longstanding phylogenetic uncertainties within *Ardisia* and its allies, including the placement of *Sadiria*, *Tapeinosperma*, *Amblyanthus*, and *Amblyanthopsis*, and revealed a middle Miocene rapid radiation in *Ardisia* and its allies. Importantly, we demonstrate that the origin of leaf nodule symbiosis coincided temporally with this radiation (~11–8 Ma). Diversification analyses further show that leaf-nodulated lineages exhibit significantly elevated speciation rates, suggesting that leaf nodule symbiosis may represent a key evolutionary innovation driving diversification.

## 1. Introduction

Plants and microorganisms have developed complex symbiotic relationships throughout long-term evolution, and such associations are widespread in nature [1]. Compared with the well-known mycorrhizal symbiosis in roots [2], phyllosphere microorganisms inhabit a far more variable atmospheric environment. These unique conditions have shaped distinct interaction patterns and ecological functions. As a result, bacterial symbiosis on leaf surfaces, as a specialized ecological niche, has increasingly drawn research attention in recent years [3,4,5,6,7].

Leaf nodulation is a typical example of phyllosphere symbiosis, which represents a morphological feature where symbiotic bacteria establish stable and persistent colonization at specific sites on plant leaves. Unlike root–microbe interactions and specialized root nodules (i.e., rhizobial nitrogen-fixing symbiosis) [8], leaf nodules housing microbial endophytes in dedicated glandular structures have only recently received attention [9]. Such leaf nodule symbionts have been reported only in a few plant lineages, e.g., *Ardisia* Sw. and its allies *Amblyanthopsis* Mez and *Amblyanthus* A.DC. (Primulaceae), several Rubiaceae genera (i.e., *Pavetta* L., *Psychotria* L. and *Sericanthe* Robbr.), *Styrax* L. (Styracaceae), and Dioscoreaceae [5,10,11,12]. Current evidence indicates that the morphology, size, and distribution of leaf nodules show a degree of species specificity [4,12]. For example, in *Psychotria* (Rubiaceae), leaf nodules are randomly distributed in the lamina, whereas in *Dioscorea sansibarensis* (Dioscoreaceae) they are restricted to the leaf apex. In bacterial symbiotic species of Primulaceae, leaf nodules are generally distributed along the leaf margin.

Significant and fascinating progress has been made in the study of leaf nodule symbiosis. First, studies have shown that leaf nodule symbionts in *Ardisia*, *Psychotria*, and *Pavetta* are *Burkholderia* belonging to the Burkholderiaceae family of β-proteobacteria, while the symbiont of *Dioscorea* Plum. ex L. (Dioscoreaceae) is *Orrella dioscoreae* [13]. Second, due to the highly specialized mutualistic relationship between leaf nodule bacteria and their hosts, *Candidatus Burkholderia* species cannot be cultivated outside their plant hosts. Conversely, host plants (such as *Psychotria kirkii* Hirkii and *Ardisia crenata* Sims) exhibit severe growth defects and eventually die when deprived of their endosymbionts [12,14,15,16]. Third, nodulated *Burkholderia* species display obligate symbiosis and are primarily transmitted vertically through seeds [3]. Additional routes have been proposed, including insect-mediated transmission [4] and pollen-mediated transfer, since *Burkholderia* spp. have been detected in the stamens of *A. crenata* [17].

Finally, host plants derive clear benefits from their leaf nodule symbionts. For example, the symbionts of *A. crenata* produce the cyclic depsipeptide FR900359, a potent and selective inhibitor of Gq proteins [18,19,20,21], which enhances host resistance against pathogens and herbivores. A similar case has been reported in Rubiaceae, where *Candidatus Burkholderia* endophytes produce the toxic alkaloid pavettamine [5,22]. These observations suggest that leaf nodule symbiosis may confer ecological advantages to host plants by facilitating niche expansion or reducing biotic pressures. A plausible hypothesis is that symbiosis promotes the diversification of host plants. However, evidence supporting this hypothesis remains limited, and more direct investigations into leaf nodule symbiosis are needed to rigorously test it. As noted above, *Ardisia* and its close relatives *Amblyanthopsis* and *Amblyanthus* are typical leaf-nodule-symbiotic plants and represent ideal examples for investigating whether this trait is linked to the formation of species diversity.

*Ardisia*, the largest pantropical genus of Myrsinoideae (Primulaceae *s.l.*), comprises approximately 700–1000 species throughout the world [23], nearly 400 of which occur in Asia. It is a characteristic component of tropical rainforests and Asian evergreen broad-leaved forests [24]. Previous studies have shown that *Ardisia*, as currently circumscribed, is not monophyletic, because several closely related genera—such as *Amblyanthopsis*, *Amblyanthus*, *Tapeinosperma* Hook.f., *Labisia* Lindl., *Parathesis* Hook.f., *Elingamita* G.T.S.Baylis, and others—are phylogenetically associated with or nested within it [6,25,26,27,28]. Given its remarkable species richness, a comprehensive and well-resolved phylogenetic framework remains lacking. Consequently, the relationships between *Ardisia* and its allied genera remain taxonomically unresolved, and it is still unclear whether *Ardisia* should be divided into several smaller genera or whether its close allies should be incorporated into a more broadly circumscribed *Ardisia*. Therefore, in the present study, we refer to the traditionally circumscribed *Ardisia* and its close allies collectively as the “Ardisioids”.

Across Primulaceae, leaf nodule symbiosis is known only in *Ardisia* subg. *Crispardisia*, *Amblyanthopsis*, and *Amblyanthus* [12]. Leaf nodule symbiosis is a key morphological trait of *Ardisia* subg. *Crispardisia* [6,12,17,18]. This subgenus is one of the most species-rich *Ardisia* groups in China and the Indo-Burma region, comprising about 36 species [29]. *Amblyanthopsis* and *Amblyanthus* also exhibit crenate leaves with nodules. They differ from *Ardisia* subg. *Crispardisia* mainly by their fused anthers and by their relative stamen filament length compared with anthers [28,30,31]. Molecular phylogenetic studies have suggested that *Amblyanthopsis* and *Amblyanthus* are nested within *Ardisia* and placed as sister to *Ardisia* subg. *Crispardisia* [28]. However, due to the lack of comprehensive sampling and richer molecular data (particularly nuclear genomic information), phylogenetic relationships among *Ardisia* and its allies are not well understood. Consequently, the origin of leaf nodule symbiosis within Primulaceae, as well as its potential evolutionary importance in driving diversification rates, remains unresolved.

In this study, we sampled extensively across China and adjacent regions, covering *Ardisia* and its close relatives (e.g., *Amblyanthopsis* and *Amblyanthus*), and included representatives of all subgenera of *Ardisia* in the area (subgenera *Crispardisia*, *Akosmos*, *Tinus*, *Bladhia*, and *Odontophylla*). Special emphasis was placed on collecting species of *Ardisia* subg. *Crispardisia*. We generated high-throughput sequencing data for each species and reconstructed phylogenetic relationships using state-of-the-art systematic methods. Specifically, we aim to (1) reconstruct the phylogeny of *Ardisia* and related genera (“Ardisioids”); (2) reconstruct its evolutionary history; and (3) evaluate the evolutionary origin of leaf nodule symbiosis of *Ardisia* and its relatives, and its potential impact on diversification rate.

## 2. Material and Methods

### 2.1. Taxon Sampling and DNA Extraction

We sampled 134 accessions, including 126 newly collected individuals. A total of 65 *Ardisia* species were sampled in this study. Specifically, it covers all five subgenera of Chinese *Ardisia* (subg. *Crispardisia*, *Akosmos*, *Bladhia*, *Tinus*, and *Odontophylla*), representing 60 of the 70 (~86%) species recorded in China [32]. Newly collected voucher specimens were formally identified by Gang Hao, Ge-Han Huang and Xiao-Kai Yan, and deposited at the Herbarium of South China Botanical Garden (IBSC), Chinese Academy of Sciences (CAS). Please find the information in Supplementary Table S1. We also included eight other genera closely related to *Ardisia*. Silica-gel-dried leaves were used for total genomic DNA extraction with a modified Cetyltrimethylammonium bromide (CTAB) protocol [33]. DNA integrity was evaluated using agarose gel electrophoresis and Qubit quantification.

### 2.2. Genome Sequencing and Plastid Assembly

Genomic libraries (2 × 150 bp) were prepared using the MGIEasy kit (MGI Tech Co., Ltd., Shenzhen, China) and sequenced on the MGISEQ-2000 platform at BGI (Wuhan, China). Raw reads were processed with Trimmomatic v0.36 [34] to remove adapters and low-quality bases. Each sample yielded ~2 Gb clean reads.

Plastid genomes were assembled with GetOrganelle [35] using the “embplant_pt” reference set and *Ardisia crenata* plastome (MW929178) as the seeds. Circularized assemblies were validated by read mapping in Geneious v11.0.4. Annotation was performed with GeSeq [36] and verified with Chloë v0.1.0 (https://github.com/ian-small/chloe—Accessed on 9 June 2025). The summary of the sequencing and plastid genome data were provided in Supplementary Table S2.

### 2.3. Concatenated Plastid Phylogeny

Eighty conserved protein-coding sequences were extracted from 134 plastid genomes according to the gene annotations and translated into amino acid sequences. Protein sequences were aligned using the MAFFT L-INS-i algorithm [37], optimized for local iterative refinement. The protein alignments were back-translated into codon alignments, and trimmed using trimAl by removing columns with >50% missing data (Supplementary Table S3). These gene alignments were used for both phylogeny and divergence time estimation.

Concatenated plastid phylogenies were reconstructed using both RAxML v8.2.13 [38] and IQ-TREE v2.4.0 [39]. With both analyses, two additional partitioning strategies were implemented: (1) partitioning by gene (80 partitions) and (2) partitioning by codon position (three partitions). In RAxML, maximum likelihood (ML) analyses were performed with the General Time Reversible (GTR) + Γ substitution model and 500 rapid bootstrap (RBS) replicates. In IQ-TREE, ModelFinder was employed to identify the best-fitting substitution models [40]. Node support was evaluated using 1000 SH-like approximate likelihood ratio (SH-aLRT) tests and ultrafast bootstrap (UFBoot) analyses with 1000 replicates, incorporating the -bnni correction to reduce potential bootstrap overestimation. Branches with SH-aLRT ≥ 80% and UFBoot ≥ 95% were considered strongly supported.

Phylogenetic trees inferred from a concatenated plastid dataset under different analytical strategies were compared using Robinson–Foulds (RF) distance. To enable direct comparison across studies with varying taxon sampling and to facilitate assessment of phylogenetic accuracy, RF distances were normalized by dividing the raw RF value by 2(n − 3), where *n* represents the number of taxa. In addition, weighted RF distances were calculated to account for branch support associated with bipartitions unique to one of the compared trees. This approach incorporates support values into the metric, effectively weighting topological differences according to their evolutionary reliability.

### 2.4. Nuclear Ribosomal DNA Assembly and Its Phylogeny 

nrDNA sequences were also assembled in GetOrganelle, with the “embplant_nr” reference dataset. Candidate high-copy contigs were aligned to the nrDNA sequence of *Maesa montana* A.DC. (KU569493), and the longest, structurally complete repeat unit (ETS-18S-ITS1-5.8S-ITS2-26S) for each sample was selected. This procedure minimizes the impact of incomplete concerted evolution among nrDNA repeats. A total of 126 nrDNA sequences were retained for downstream analyses (Supplementary Table S1). Sequences were aligned using the MAFFT L-INS-i algorithm, and poorly aligned regions were removed at both ends. A maximum likelihood tree was constructed in RAxML with the GTR + Γ model and 500 rapid bootstrap replicates.

### 2.5. Whole-Genome-Wide Variant Calling and Species Tree Reconstruction

Compared with the relatively conserved nrDNA region, genome-wide SNP data provide substantially higher resolution for phylogenetic inference and have become an important approach in evolutionary studies. WASTER (Without Alignment/Assembly Species Tree EstimatoR) is a powerful de novo method for inferring shallow species trees directly from short-read data [41]. It employs a k-mer-based strategy that enables species tree reconstruction from low-coverage genomic reads without requiring genome assembly or sequence alignment. In this study, clean reads from sequencing were decomposed into 19 bp k-mers, which were subsequently clustered for downstream analyses. A cluster was retained only if all species shared a single variable site located at the central position of the k-mer. Clusters containing within-species polymorphisms were discarded, thereby retaining only homozygous variants and eliminating heterozygous sites. The resulting set of variant sites was concatenated and supplied to CASTER (Coalescence-aware Alignment-based Species Tree EstimatoR)-site for species tree inference under the T92 substitution model, which is compatible with strand-agnostic sequencing. Branch supports are measured as local bootstrap support (>95.0 means highly supported).

### 2.6. Heterogeneity in Phylogenetic Signals Among Plastid Genes

Although plastid protein-coding genes are typically assumed to share a common evolutionary history, substantial heterogeneity in phylogenetic signals among genes can still occur. To quantify gene tree concordance and conflict across plastid loci, we employed a gene tree-based approach using PhyParts [42]. Individual plastid gene trees were inferred using RAxML under the same parameter settings as described for the nrDNA analyses. Branches with RBS values below 50% were collapsed to reduce the impact of poorly supported relationships.

The resulting gene trees were then compared with the reference topology (the concatenated plastid ML tree) using PhyParts. This approach decomposes each internal node of the reference tree and quantifies the number of gene trees that (1) support the focal bipartition, (2) conflict with the current topology, or (3) are uninformative. The distribution of concordant and conflicting signals across nodes was visualized using pie charts.

### 2.7. Divergence Time Estimation

Divergence times were estimated using MCMCTree in the PAML v4.9 package [43], based on a dataset of 80 concatenated PCGs. This tree was rooted by designating the two species of *Maesa* Forssk. (Maesoideae) as the outgroups. Fossils and secondary calibration points were then applied to the rooted topology (Supplementary Table S4).

We estimated divergence times using the approximate likelihood method in MCMCTree. BASEML was first used to obtain preliminary substitution-rate estimates, which were then used in MCMCTree to derive maximum likelihood branch lengths along with the corresponding gradient and Hessian. A Taylor-approximated likelihood was subsequently constructed, and MCMCTree was run to obtain posterior estimates of node ages and associated parameters. Convergence was assessed in Tracer, ensuring ESS values > 200. The resulting posterior distributions were summarized to generate the final chronogram with 95% credibility intervals.

### 2.8. Diversification Rate Analysis

Lineage-through-time (LTT) plots were generated using phytools [44] based on the ultrametric time tree of the subfamily Myrsinoideae and “Ardisioids.” We then simulated 300 trees under a pure-birth model and compared their LTT curves with that of the empirical time tree. In addition, diversification rates and possible rate shifts across Old World *Ardisia* (including *Amblyanthus* and *Amblyanthopsis*) were further inferred using BAMM v2.5 [45]. Before that, priors were determined using the R package BAMMtools v2.1.6 [45]. The prior for “ExpectedNumberofShifts” was set to 1, and the global sampling probability was set to 0.18. We ran BAMM for 2,000,000 generations, sampling every 200 generations. We then evaluated convergence based on ESS ≥ 200 for the loglikelihood and number of shifts using the R package CODA v0.19-4.1 [46]. We discarded the first 25% of BAMM output files as burn-in. Using BAMM posterior probability, we inferred the 95% credible set of macroevolutionary rate shift configurations. We then plotted speciation rates, extinction rates, and net diversification rates using the R package BAMMtools.

### 2.9. State-Dependent Diversification Rates Analysis

We used the Hidden State Speciation and Extinction (HiSSE) framework to evaluate whether the presence or absence of leaf nodules is associated with diversification rates in *Ardisia*. We compared five models differing in the way they account for trait dependence and diversification rate heterogeneity. First, we fitted the standard Binary State Speciation and Extinction (BiSSE) model, which assumes that diversification rates differ directly according to the observed binary trait (presence vs. absence of leaf nodules). Second, we applied two trait-dependent HiSSE models incorporating unobserved rate heterogeneity: HiSSE2 (with two hidden rate classes) and HiSSE4 (with four hidden rate classes). Finally, to test whether diversification rate variation can be explained by unmeasured factors alone, we included character-independent diversification models, which allow two character-independent (CID-2) or four character-independent (CID-4) hidden states but constrain diversification to be independent of the focal trait. All models were implemented in RevBayes [47], and model fit was compared using marginal likelihoods estimated via stepping-stone sampling.

## 3. Results

### 3.1. Phylogenomics of Ardisia and Its Allies Based on Plastid and Nuclear Datasets

This study generated a plastid dataset comprising 80 protein-coding genes (PCGs), totaling 68,964 bp (Supplementary Table S3). The dataset includes 134 plastid genome sequences, representing 65 *Ardisia* species, 24 samples from 12 closely related genera within Myrsinoideae, and two outgroups belonging to Maesoideae. We also generated an nrDNA dataset and a genome-wide SNP dataset. Detailed information is provided in Supplementary Table S1.

**Figure 1:**
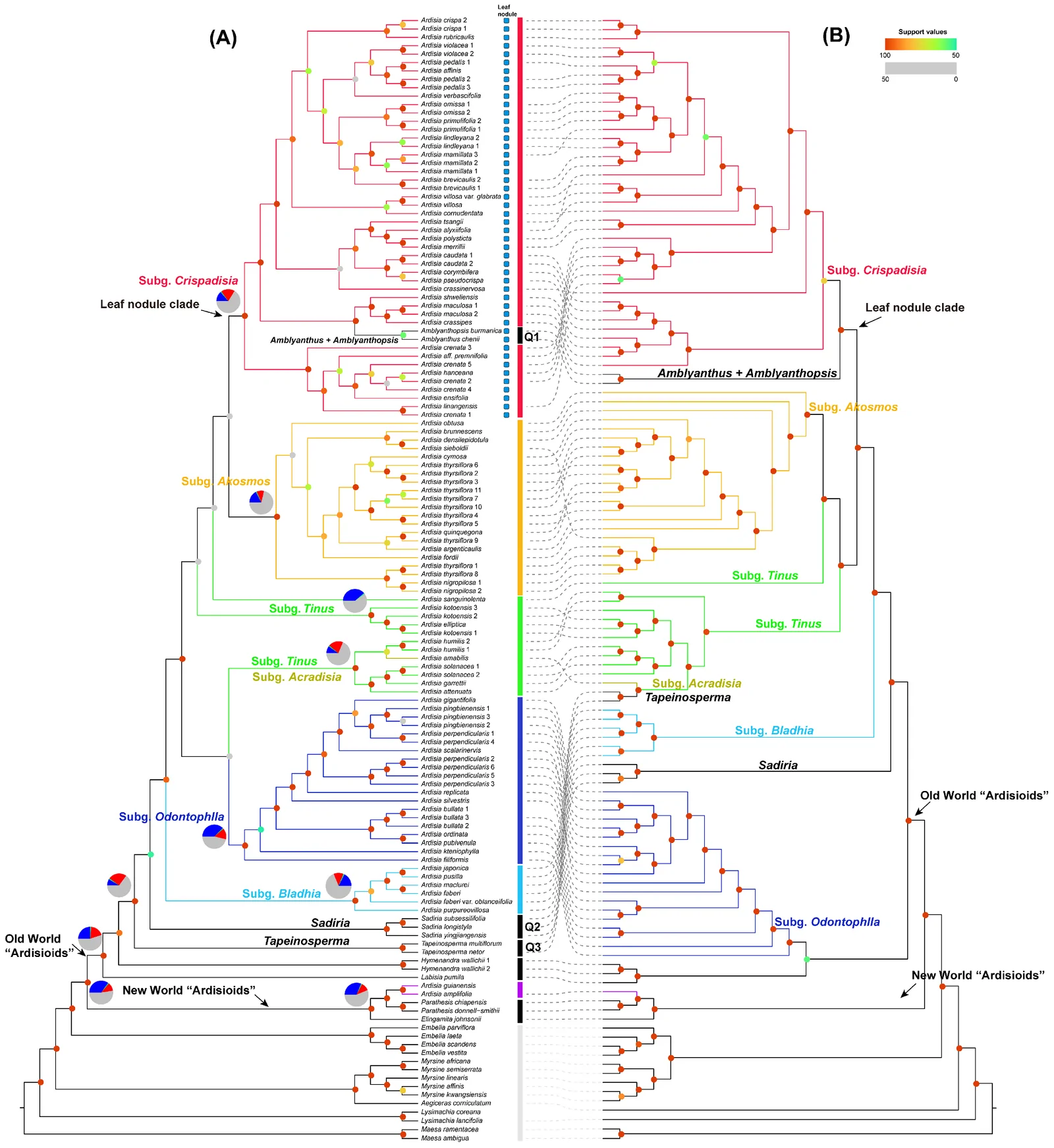
Phylogenetic trees of “Ardisioids”. (**A**) A cladogram inferred from concatenated plastid protein-coding sequences using RAxML. PhyParts results are shown at selected nodes representing recognized subgenera or genus-level clades (see Supplementary Figure S6 for details). (**B**) A species tree inferred from a genome-wide SNP dataset using WASTER. Bootstrap support values or local bootstrap support for both trees are shown above corresponding branches using colored circles. Colored circles at internal nodes represent support values based on the scale (top right), with major clades indicated. Vertical black bars (Q1–Q3) mark key topological incongruences between datasets, specifically regarding the phylogenetic placements of *Amblyanthus* + *Amblyanthopsis* within *Ardisia* subg. *Crispadisia* (Q1), *Sadiria* (Q2), and *Tapeinosperma* (Q3).

The plastid dataset was used to reconstruct phylogenetic trees under the maximum likelihood framework implemented in RAxML and IQ-TREE. The normalized RF (nRF) and weighted nRF distances were both less than 3% and 1%, respectively (Supplementary Table S5), indicating a high degree of topological congruence among ML trees inferred under different partitioning strategies. Direct comparisons of tree topologies further show that the observed discrepancies are confined to nodes with bootstrap support below 50% (Supplementary Figures S1–S5). Consistently, PhyParts analyses demonstrate that these poorly supported nodes lack concordant phylogenetic signals (Supplementary Figure S6). We therefore selected the unpartitioned topology as the representative plastid phylogeny for subsequent analyses (Figure 1A, Supplementary Figure S1). The species tree inferred from genome-wide SNP data using WASTER is shown in Figure 1B. Because nrDNA sequences are relatively conserved, the resulting topology provides limited phylogenetic resolution; therefore, this result is presented in the Supplementary Materials (Supplementary Figure S7).

All of our phylogenomic analyses indicate that the current circumscription of *Ardisia* is clearly not monophyletic (Figure 1, Supplementary Figures S1–S5). Two New World *Ardisia* species (*Ardisia amplifolia* Standl. and *A. guianensis* (Aubl.) Mez) were included in this study. However, only *A. amplifolia* was incorporated into the nrDNA and genome-wide SNP phylogenetic trees, as raw sequencing data for *A. guianensis* were unavailable. The phylogenetic results nonetheless strongly support a well-resolved clade comprising New World *Ardisia* taxa together with the New World genus *Parathesis* and the New Zealand genus *Elingamita* (Figure 1B, Supplementary Figures S7 and S8). This New World clade is in turn recovered as the sister group to the Old World “Ardisioids” lineages. The Old World *Ardisia* is a moderately well-supported group that contains two small genera, *Amblyanthus* and *Amblyanthopsis*, based on plastid genes (Figure 1A), while it further contains the genera *Hymenandra* A.DC. ex Spach, *Sadiria* Mez, and *Tapeinosperma* in the species tree recovered by a genome-wide SNP dataset (Figure 1B). The status of *Labisia* clusters with *Hymenandra* was strongly recovered in the species tree (Figure 1B).

Within Old World *Ardisia*, three well-supported monophyletic subgenera were recovered by all three datasets: subg. *Akosmos*, subg. *Bladhia*, and subg. *Odontophylla*, which could be supported by a number of concordant phylogenetic signals on the corresponding nodes inferred by PhyParts (Figure 1A). Both plastid and nrDNA phylogenies indicate that *Ardisia* subg. *Crispardisia* is paraphyletic, as *Amblyanthus* and *Amblyanthopsis* are nested within it (Figure 1A, Supplementary Figure S7). However, the species tree inferred from genome-wide SNPs supports that *Ardisia* subg. *Crispardisia*, together with *Amblyanthus* and *Amblyanthopsis,* forms a distinct clade, although the relationship among these three genera remains poorly resolved (Figure 1B, Supplementary Figure S8). Furthermore, all of our datasets consistently show that *Ardisia* subg. *Tinus* is polyphyletic, with its species distributed across two distinct clades (Figure 1, Supplementary Figures S1–S5, S7 and S8), and *Ardisia* subg. *Acrardisia* consistently nested within it. In addition, *Tapeinosperma* was further resolved as sister to *Ardisia* subg. *Acrardisia* in the species tree (Figure 1B, Supplementary Figure S8).

In addition, some taxonomically challenging groups, characterized by pronounced morphological variation, remain unresolved. Notably, the *Ardisia crenata* complex (i.e., *A. crenata*, *A. henceana* Mez, *A. linanensis* C.M.Hu, and *A. ensifolia* E.Walker) and the *Ardisia thyrsiflora* complex (i.e., *A. thyrsiflora* D.Don, *A. cymose* Blume, and *A. quinquegona* Blume) continue to present difficulties. Within these complexes, *A. crenata* and *A. thyrsiflora* exhibit exceptionally broad geographic and elevational ranges and substantial morphological variability [32,48]. 

### 3.2. Cytonuclear Conflicts

Many nodes in the nrDNA ML tree showed bootstrap support values below 50 (Supplementary Figure S7); therefore, we primarily focused on comparing the plastid phylogeny with the genome-wide SNP species tree. It is evident that substantial cytonuclear conflicts exist between these two phylogenies (Figure 1). Here, we specifically highlight three major conflicts: (1) the phylogenetic placement of *Amblyanthus* and *Amblyanthopsis* (Q1); (2) the phylogenetic position of *Sadiria* (Q2); and (3) the phylogenetic position of *Tapeinosperma* (Q3) (Figure 1).

### 3.3. Divergence Time Estimate of “Ardisioids”

**Figure 2:**
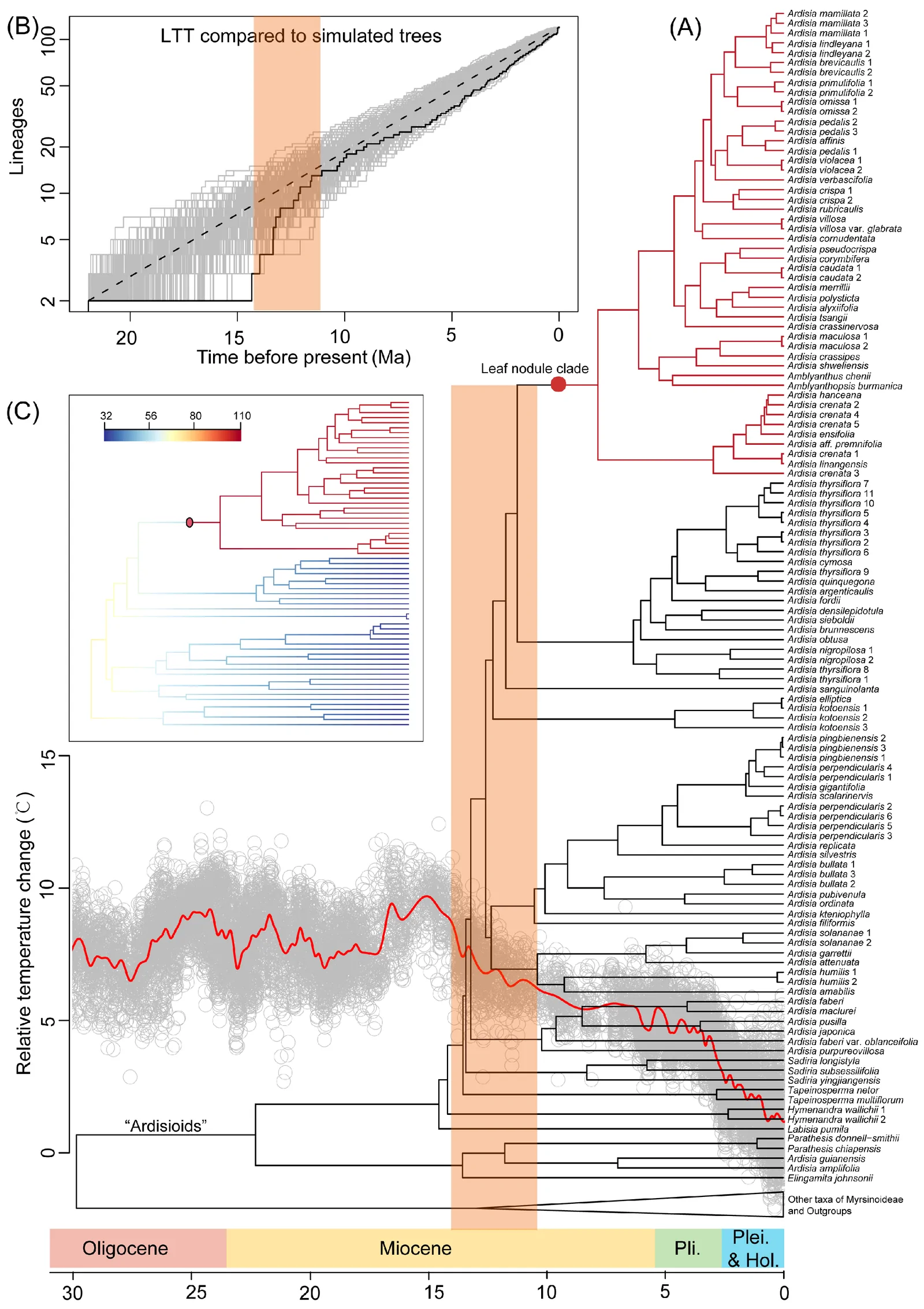
A chronogram, LTT plot and Phylorate analysis of “Ardisioids”. (**A**) The chronogram generated using MCMCTREE features yellow shading marking a period of rapid radiation, with Cenozoic temperature changes (red) adapted from Westerhold et al. [49]. (**B**) Phytools-inferred LTT plot of “Ardisioids,” with yellow shading denoting the period of rapid lineage accumulation. (**C**) Phylorate plot showing variation in speciation rates across the phylogeny of Old World *Ardisia*, inferred with BAMM.

We reconstructed the evolutionary history of the subfamily Myrsinoideae based on 80 plastid genes, revealing that its early diversification can be traced back to approximately 49 million years ago (Ma), corresponding to the early Eocene. “Ardisioids” is estimated to have originated around 29 Ma (stem age) and subsequently split into Old World and New World lineages at approximately 22 Ma. Our results further indicate that diversification of the New World “Ardisioids” began around 14 Ma. At roughly the same time, Old World “Ardisioids” also initiated a rapid radiation, giving rise to at least five genera—*Labisia*, *Hymenandra*, *Tapeinosperma*, *Sadiria*, and *Ardisia* (Figure 2A). The presence of numerous short internodes during the early diversification of the Old and New World “Ardisioids” also indicates a phase of rapid lineage splitting (Supplementary Figures S1–S5 and S7). The PhyParts analysis indicated that most genes lacked concordant phylogenetic signals during the period (Supplementary Figure S6). Interspecific divergence within Old World *Ardisia* occurred since the Quaternary. The *Ardisia crenata* complex exemplifies this pattern of recent and rapid diversification (Figure 2A). Importantly, lineages exhibiting leaf nodulation show a stem age of approximately 11 Ma and a crown diversification age of around 8 Ma.

### 3.4. Diversification Rate

The LTT plot for the entire subfamily Myrsinoideae reveals a pronounced increase in lineage accumulation between 14 and 12 Ma (Figure 2B), coinciding with a phase of rapid lineage splitting during the early diversification of both Old and New World “Ardisioids” (Supplementary Figures S1–S6). Phylorate analysis detected a single, well-supported rate shift within the leaf nodule clade (i.e., *Ardisia* subg. *Crispardisia* + *Amblyanthus* + *Amblyanthopsis*), dated to approximately 10 Ma (Figure 2C).

**Figure 3:**
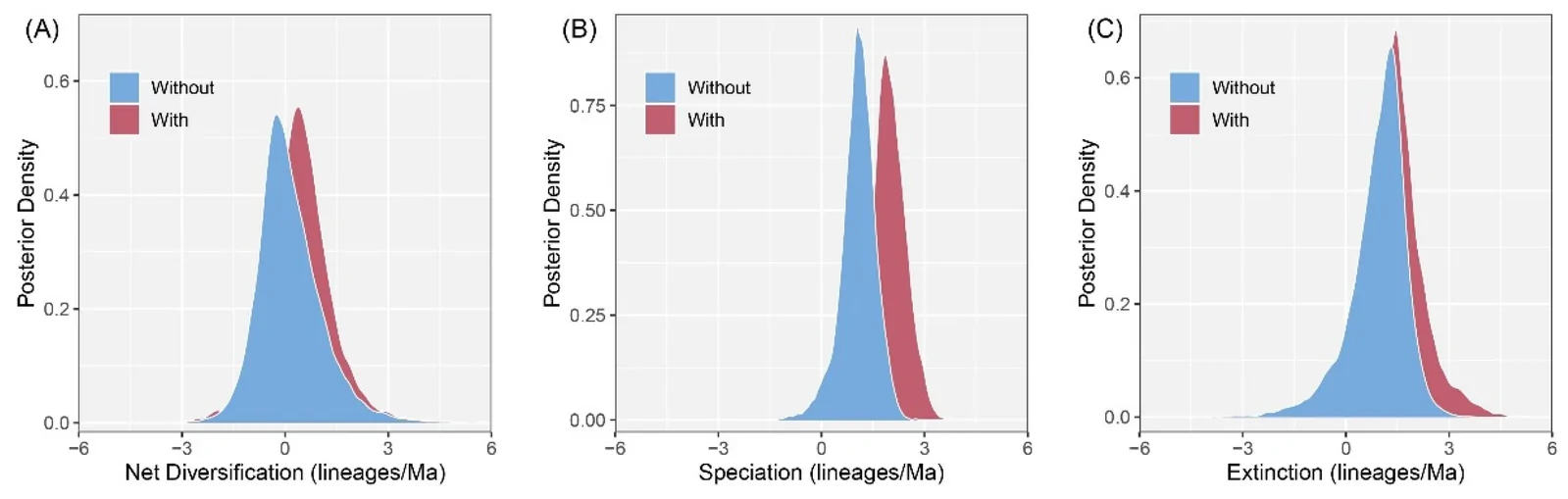
Posterior distributions of speciation, extinction, and net diversification rates for *Ardisia* lineages with and without leaf nodules under the best-fitting HiSSE4 model. Lineages with leaf nodules are shown in red and their non-nodule relatives in blue. The subfigure (**A**) Shows the posterior density distribution of net diversification rates (speciation–extinction); (**B**) Displays the posterior density of speciation rates; (**C**) Compares the posterior density of extinction rates.

We further investigated whether the leaf nodulation trait is associated with diversification rates. Model comparison based on marginal likelihoods indicated that the HiSSE4 model best explained the data (Supplementary Table S6), outperforming both the simpler BiSSE and the character-independent CID models. This indicates that diversification rate variation in *Ardisia* is shaped by a combination of the leaf nodule and additional, unmeasured factors. Under the best-fitting HiSSE4 model, lineages with leaf nodules exhibited consistently higher net diversification rates (r = λ − µ) than their non-nodule relatives (Figure 3). This pattern was primarily driven by elevated speciation rates (λ1) in nodule-bearing lineages, while extinction rates (µ0 and µ1) were comparable between the two groups (Figure 3). These results suggest that the presence of leaf nodules is positively associated with diversification in *Ardisia*, primarily through enhanced speciation rather than reduced extinction (Figure 3). 

## 4. Discussion

### 4.1. Traditional Delimitation of Ardisia and Its Relatives Needs to Be Re-Evaluated

Traditionally, *Ardisia* has been recognized as the largest genus within Myrsinaceae *s.s*, and even after its reclassification under the broadly defined Primulaceae, it remains the largest genus in the family. Due to its extraordinary species richness and pronounced morphological diversity, resolving its phylogenetic relationships with closely related genera and establishing a robust infrageneric classification system have proven to be significantly challenging.

Our study, in line with previous phylogenetic analyses, consistently indicates that *Ardisia*, as currently circumscribed, is not monophyletic [25,26]. Broadly, it can be divided into two major geographically defined clades: (1) a New World clade, comprising American *Ardisia* taxa and their allied genera (e.g., *Elingamita, Wallenia* Sw., *Parathesis*, *Geissanthus* Hook.f., and *Stylogyne*) from the Americas, and (2) an Old World clade (e.g., *Badula* Juss., *Oncostemum* A.Juss, *Amblyanthus*, *Amblyanthopsis*, *Discocalyx* (A.DC.) Mez, *Fittingia* Mez, *Antistrophe* A.DC., *Conandrium* (K.Schum.) Mez, *Labisia*, *Systellantha* B.C.Stone, *Sadiria*, and *Loheria* Merr.), which spans a wide distribution across Asia, the Atlantic islands, Australia, and Africa [6,25,26,50].

However, our sampling in this study was largely concentrated in Asia, particularly on *Ardisia* taxa from China and adjacent regions. In contrast, only a few New World *Ardisia* species were included, and the type species of the genus was not sampled. Moreover, *Ardisia* and its close allies comprise numerous taxa, and in the absence of broader phylogenetic analyses, no formal taxonomic treatment has yet determined whether these allied genera should be incorporated into *Ardisia*. Therefore, accurately redefining the generic boundaries of *Ardisia* and fully resolving its taxonomic issues lies beyond the scope of this study. Instead, our primary focus is on clarifying the phylogenetic relationships within *Ardisia* from China and surrounding areas, and examining the systematic placement of several of its closest allies.

#### 4.1.1. The Phylogenetic Position of *Tapeinosperma*

Based on current evidence, the New Zealand endemic group *Elingamita* appears to have a closer phylogenetic relationship with the Neotropical clade [26,27]. More intriguingly, the phylogenetic position of *Tapeinosperma*, distributed across Southeast Asia and the Pacific Islands, presents a particularly interesting case. Given its geographical distribution, this group would presumably belong to the Old World clade of *Ardisia*, which was strongly supported by our phylogenetic analyses (Figure 1) and by Rose et al. [27]. However, recent studies based on nuclear genes (the Angiosperms353 probe set) present a different perspective: the two included *Tapeinosperma* species (i.e., *T. pseudojambosa* (F.Muell.) Mez and *T. storezii* M.Schmid) were strongly supported as members of the Neotropical clade, forming a sister group to *Elingamita* [26,51].

Morphologically, *Tapeinosperma* species are characterized by cylindric styles and conspicuous stigmas, which clearly distinguish them from *Ardisia*; however, they are not easily separable from their close relative *Discocalyx* [52]. The discordance in the phylogenetic placement of *Tapeinosperma* may be attributed to two possible explanations. First, *Tapeinosperma* may in essence not be monophyletic, with some species more closely related to *Elingamita* and the New World “Ardisioids,” while others are aligned with the Old World “Ardisioids”. Yet, this explanation seems highly implausible, because studies—including our own—that randomly sampled six distinct species of *Tapeinosperma* consistently recovered the genus as monophyletic in both nuclear and plastid datasets [26,27,51]. Second, the observed discordance may reflect ancient episodes of extensive hybridization and introgression, which is currently considered the most common cause of cytonuclear conflict [53]. Nevertheless, the striking discrepancies between genome-wide SNPs (Figure 1B) and nrDNA (Supplementary Figure S7) and those derived from the Angiosperms353 probe set underscore the need for further detailed investigation into both the phylogenetic placement and the taxonomic circumscription of *Tapeinosperma*.

#### 4.1.2. The Phylogenetic Position of *Sadiria*

*Sadiria* is a small, well-defined genus established by Mez [31]. It resembles *Ardisia* but differs in having petals united to or above the middle and flowers borne in very short (equal to or shorter than the petioles), axillary, subfasciculate cymose or subpaniculate inflorescences [31,54]. The genus comprises nine species and two varieties, distributed from the eastern Himalaya and Khasi Hills to northern Myanmar and southern Yunnan, China [31,54,55,56,57,58,59].

Our results reveal pronounced cytonuclear discordance. Plastid phylogenomic analyses strongly support three *Sadiria* species (including *S. longistyla* Ze H.Wang & H.Peng) forming a clade that is sister to Old World *Ardisia* (Figure 1A). In contrast, nuclear data consistently place *Sadiria* within Old World *Ardisia*, most closely related to *Ardisia* subg. *Tinus* or *Ardisia* subg. *Bladhia* (Figure 1B, Supplementary Figure S7), which is supported by Julius et al. [25] and Larson et al. [26].

Cytonuclear discordance, increasingly recognized across plant lineages, often reflects complex evolutionary histories such as ghost lineages, hybridization, or introgression [60,61]. Careful re-examination of voucher specimens ruled out misidentification. We therefore infer that the observed phylogenetic conflict most likely resulted from ancient hybridization or introgression between *Sadiria* and *Ardisia*. The plastid genome of *Sadiria* likely represents a ghost lineage inherited from a hybrid ancestor. These findings suggest that *Sadiria* could potentially be treated as a subgenus within *Ardisia*. However, because the type species of *Ardisia* is Neotropical, and molecular evidence clearly separates New World and Old World *Ardisia* into distinct clades associated with geographically proximate genera, any taxonomic revision would need to be considered at the genus-wide level.

#### 4.1.3. The Phylogenetic Position of *Ardisia* subg. *Crispardisia*, *Amblyanthus* and *Amblyanthopsis*

*Ardisia* subg. *Crispardisia* can be recognized by its crenulate leaves bearing marginal nodules, which clearly distinguish it from other *Ardisia* [29,31,59,62]. The subgenus comprises about 36 species [29,32], primarily distributed in subtropical and tropical regions of China and Indo-Burma. *Amblyanthus* and *Amblyanthopsis* are morphologically similar to *Ardisia*, especially because both also possess crenate leaves resembling those of *Ardisia* subg. *Crispardisia*, but they are distinguishable by specific traits. For example, anthers in *Amblyanthus* are laterally connate into a cone, while in *Amblyanthopsis*, they are coherent but not fused [31,59]. Each of these two small genera contains five species: *Amblyanthus* occurs in Bangladesh, northeastern India, and southwestern China [28,31,58,59], whereas *Amblyanthopsis* is distributed in Bangladesh, Bhutan, Myanmar, and northeastern India [30,63]. The monophyly of *Ardisia* subg. *Crispardisia* has been supported in earlier studies [6,17,25], but its status remains questionable because of limited sampling and omission of its presumed close relatives (*Amblyanthus* and *Amblyanthopsis*). In this study, for the first time, we included 33 of the 36 species of *Ardisia* subg. *Crispardisia*, along with two species representing *Amblyanthus* and *Amblyanthopsis*. Nuclear and plastid genomic data allowed us to make several observations.

Our analyses clearly indicate that the three groups are closely related and further demonstrate that *Amblyanthus* and *Amblyanthopsis* are sister taxa. Their close affinity had also been suggested by Zhou et al. [28]. From this, we can at least conclude that anther connation is not an ideal character for distinguishing *Ardisia* from its close relatives. For example, the fused anthers of *Amblyanthus* are not unique but also occur in other genera such as *Conandrium* and *Hymenandra* [25,59], with phylogenetic analyses indicating multiple independent origins. If our findings are correct, the presence of leaf nodules likely represents a single evolutionary origin within Primulaceae. All three groups (*Ardisia* subg. *Crispardisia*, *Amblyanthus*, and *Amblyanthopsis*) have petiolate leaves with glandular-crenate margins, identical to the condition in *Ardisia* subg. *Crispardisia*. These marginal glands were shown to be leaf nodules, representing an obligate bacterial leaf symbiosis essential for normal host growth [3,6,11,12]. Our divergence time estimates suggest that this symbiosis originated around 11 Ma (stem age) to 8 Ma (crown age), approximately three million years earlier than estimated by Lemaire et al. [3]. This discrepancy is most likely attributable to our denser sampling, differences in calibration points, or variation in analytical methods.

In addition, we found that the positions of *Ardisia* subg. *Crispardisia*, *Amblyanthus,* and *Amblyanthopsis* show cytonuclear discordance. Our whole-genome SNP-based species tree also demonstrated that *Ardisia* subg. *Crispardisia* is closely related to *Amblyanthus* and *Amblyanthopsis* (Figure 1B). However, plastid evidence strongly supports that *Amblyanthus* and *Amblyanthopsis* are closely related to *Ardisia maculosa*, *A. hokoensis*, and *A. shweliensis* within *Ardisia* subg. *Crispardisia*, suggesting plastid capture from either extinct or undescribed *Ardisia* subg. *Crispardisia* taxa.

However, there remains disagreement as to whether the systematic position of *Amblyanthus* is indeed closely associated with *Ardisia* subg. *Crispardisia* and *Amblyanthopsis*. For example, Julius [64], in her doctoral dissertation, found that *Amblyanthus* did not form a sister relationship with these two taxa, suggesting that leaf nodulation may not have a monophyletic origin. This result therefore warrants caution regarding the true phylogenetic placement of *Amblyanthus*. Indeed, our study included only a single species of *Amblyanthus*; broader taxon sampling will be required to clarify its phylogenetic position in future studies.

### 4.2. Rapid Divergence Events Within the Old World Ardisia and Its Relatives

Despite the large amount of data used in this study—80 plastid genes and genome-wide SNP datasets—many nodes in the inferred phylogeny still received very low support. Specifically, on the ML phylogenetic trees constructed from plastid genomes and nrDNA, the majority of these nodes lacked significant support (BP < 70, SH-aLRT < 80%, and UFBoot < 95%) (Supplementary Figures S1–S5 and S7). We further found that these weakly supported nodes mainly occurred in two regions of the tree: (1) Among relationships between subgenera within *Ardisia* and between *Ardisia* and its close relatives (i.e., *Tapeinosperma* and *Sadiria*), where the branches were also extremely short. The very short branch lengths observed here indicate a lack of phylogenetic signal, consistent with a rapid diversification event [65]. (2) Low support was also observed in many species pairs, particularly within *Ardisia* subg. *Crispardisia*, where at least 12 nodes lacked significant support and were likewise characterized by short branches. All topological evidence above is visualized in Figure 1A and Supplementary Figures S1–S5 and S7.

Our molecular dating analyses suggest that the first episode of rapid radiation occurred during the middle Miocene, approximately 14–13 Ma. A similar event was also reported by Larson et al. [26]. From the early Miocene, Southeast Asia experienced a climatic shift from seasonally wet to predominantly perhumid conditions [66]. During the Middle Miocene Thermal Maximum, megathermal forests expanded northward to southern China (ca. 24° N), fostering a diverse evergreen rainforest flora dominated by tropical lineages such as Dipterocarpaceae, Leguminosae, and Clusiaceae [66,67,68,69]. Previous historical biogeographic analyses (e.g., Larson et al. [26]) inferred that the ancestral area of the “Ardisioids” (including *Ardisia* and its allies) was the Indo-Malayan region. This suggests that the warmer climate and sea-level rise during this period [70] may have promoted geographic isolation among Southeast Asian islands, thereby facilitating the rapid diversification of ancestral “Ardisioid” lineages—a pattern documented in several other plant groups [70,71].

Another episode of rapid radiation appears within the lineage comprising *Ardisia* subg. *Crispardisia,* together with *Amblyanthus* and *Amblyanthopsis*. In particular, nearly all species pairs within this clade diverged only within the last 2–3 million years (since the Pleistocene). Compared with the entire genus *Ardisia*, which contains more than 700 species [23], this plant group comprises only about 45 species (36 in *Ardisia* subg. *Crispardisia*, four in *Amblyanthopsis*, and five in *Amblyanthus*) [28,29,30,31], reflecting relatively modest species richness. Nevertheless, its main distribution lies in tropical and subtropical China and adjacent regions, near the northern edge of the whole genus’ broader range. For instance, in China alone, *Ardisia* subg. *Crispardisia* contains 34 species, accounting for nearly half of the country’s ~70 *Ardisia* species, making it the most species-rich subgenus of *Ardisia* in this region. The distribution pattern highlights both the strong environmental adaptability and the relatively rapid diversification of the lineage in tropical and subtropical China.

Climatic fluctuations since the Pleistocene likely accelerated divergence and speciation in Southeast Asia. These patterns are linked to complex geological events, such as rapid orogenesis from micro-terrane movements and major sea-level shifts driven by climate oscillations [70,72], as seen in *Impatiens* Riv. ex L. [73] and *Begonia* L. [74]. The case of *Ardisia* subg. *Crispardisia* may differ from these island-related scenarios. This lineage is concentrated in tropical and subtropical China and nearby regions, at the northern margin of *Ardisia*’s Asian distribution, which hosts many endemic and rapidly radiating plant groups (such as *Ilex* Tourn. ex. L., *Actinidia* Lindl., *Lysimachia* Tourn. ex. L. and *Magnolia* sect. *Michelia* [75,76,77,78]). Their diversification may have been coupled with and driven by the uplift of the Qinghai–Tibetan Plateau, the East Asian monsoon, and global climate shifts [78]. We therefore hypothesize that *Ardisia* subg. *Crispardisia* was also strongly shaped by these external environmental factors.

### 4.3. Leaf Nodulation as a Key Innovation Driving Diversification

The leaf-nodulated clade (i.e., *Ardisia* subg. *Crispardisia* and taxa from *Amblyanthopsis* and *Amblyanthus*) exhibits a significantly elevated speciation rate (Figure 2C). Character-dependent diversification models, particularly under the robust HiSSE framework, consistently outperformed alternative models (Figure 3). These results clearly indicate that lineages possessing this intimate mutualism have experienced significantly higher speciation rates than their non-symbiotic relatives. Collectively, the evidence supports leaf nodule symbiosis as a key innovation that likely triggered radiation within the species-rich, leaf-nodulated clade of *Ardisia*.

This finding has additional significance in a broader comparative context. Similar studies in Rubiaceae have revealed a positive association between bacterial leaf endophytes and increased host diversification, including in lineages lacking distinct nodules [79]. Our results provide the first explicit test of symbiosis-driven diversification within *Ardisia*, suggesting that inherited microbial partnerships may be an underappreciated driver of plant macroevolution. These associations can fundamentally reshape host evolutionary trajectories by introducing novel functional capacities that promote ecological expansion and lineage diversification.

While identifying a statistical correlation between symbiosis and diversification is essential, understanding the underlying mechanisms is equally critical. In the *Ardisia–Burkholderia* system, a likely driver is the production of novel chemical defenses by the bacterial symbiont. In *A. crenata*, the endosymbiont synthesizes FR900359, a potent broad-spectrum defensive compound [4,6,18,19,20,21], directly linking symbiosis to host fitness.

This “borrowed” biochemical arsenal may promote diversification through multiple, non-exclusive mechanisms. First, enhanced defense could facilitate escape from herbivores and pathogens, allowing ancestral *Ardisia* subg. *Crispardisia* lineages (including taxa from *Amblyanthopsis* and *Amblyanthus*) to colonize previously inaccessible niches. Second, geographic variation in enemy communities may impose divergent selection on symbiotic defense strategies, promoting ecological specialization and ultimately reproductive isolation through allopatric or ecological speciation.

Beyond defense, the symbiosis is also essential for host growth and development. In the absence of symbionts, seedlings can survive for up to three years but remain severely stunted; the shoot tips eventually degenerate into callus tissue, and growth ceases [12,14,16]. The symbiosis may also enhance tolerance to abiotic stressors, including drought, salinity, and nutrient-poor soils [80], functioning analogously to legume–rhizobial associations [81]. By expanding the host’s fundamental niche, the partnership could facilitate broader geographic distributions, increasing opportunities for population isolation and subsequent divergence.

## 5. Conclusions

Our study focuses primarily on *Ardisia* and its close relatives (subfamily Myrsinoideae) distributed in China and adjacent regions. Through relatively comprehensive taxon sampling and by integrating plastid genome data with nuclear gene datasets, we reconstructed a robust phylogenetic framework and elucidated the evolutionary history of this group. Our results affirm that *Ardisia* is not monophyletic, but instead represents the core lineage of both Old World and New World “Ardisioids”, highlighting that generic boundaries within “Ardisioids” require more detailed taxonomic and systematic reassessment.

Within our focal groups (*Ardisia* and allied genera from China and surrounding regions), we detected a rapid radiation event during the middle Miocene, which gave rise to numerous morphologically distinct lineages. A further increase in net diversification rates was observed in lineages characterized by leaf nodule symbiosis, namely *Ardisia* subg. *Crispardisia*, *Amblyanthopsis*, and *Amblyanthus*. Our results provide strong evidence that leaf nodule symbiosis in *Ardisia* has promoted species diversification by elevating speciation rates, while extinction rates remained comparable to those of non-symbiotic lineages. This dynamic has contributed to both increased species richness and enhanced ecological adaptability within the group. Nevertheless, we cannot exclude the possibility that at least four additional, yet unidentified traits—acting independently or synergistically—have also contributed to the elevated diversity of the leaf-nodulated clade. These findings underscore the need for further research to uncover these hidden traits and to more fully reconstruct the evolutionary history of this remarkable lineage.

## Data Availability

The data is available in ScienceDB (https://doi.org/10.57760/sciencedb.35368).

## Supplementary Materials

The following supporting information can be downloaded at: https://www.mdpi.com/article/10.3390/biology15171451/s1, Figure S1: Maximum likelihood (ML) tree of “Ardisioids” based on unpartitioned plastid genes. The phylogeny was inferred using RAxML based on the concatenated 80 plastid-gene alignment matrix under an unpartitioned model (no-partition). Numbers at the nodes represent bootstrap support values derived from 500 replicates; Figure S2: Maximum likelihood (ML) tree of “Ardisioids” based on plastid data partitioned by codon position. The phylogeny was inferred using RAxML with the dataset partitioned according to the codon positions (1st, 2nd, and 3rd) of the protein-coding genes. Numbers at the nodes represent bootstrap support (BS) values derived from 500 replicates; Figure S3: Maximum likelihood (ML) tree of “Ardisioids” based on plastid data partitioned by gene. The phylogeny was inferred using RAxML with a gene-based partitioning scheme, where each gene was treated as an independent partition. Numbers at the nodes represent bootstrap support values derived from 500 replicates; Figure S4: Maximum likelihood (ML) tree of “Ardisioids” based on plastid data partitioned by codon position using IQ-TREE. The phylogeny was inferred using IQ-TREE with a codon-position partitioning scheme. The numbers above and below the nodes represent ultrafast bootstrap (UFB) supports and SH-aLRT values derived from 1000 replicates; Figure S5: Maximum likelihood (ML) tree of “Ardisioids” based on plastid data partitioned by gene using IQ-TREE. The phylogeny was inferred using IQ-TREE with a gene-based partitioning scheme. The numbers above and below the nodes represent ultrafast bootstrap (UFB) supports and SH-aLRT values derived from 1000 replicates; Figure S6: PhyParts pie charts showing gene tree conflict and concordance. The pie charts at each node summarize the level of agreement and conflict among individual gene trees compared to the reference topology (the concatenated plastid ML tree). Blue: Proportion of gene trees supporting the species tree topology. Green: Proportion of gene trees supporting the most common alternative topology. Red: Proportion of gene trees supporting all other conflicting topologies. Gray: Proportion of gene trees that are uninformative for that node. The numbers above and below the nodes represent the number of concordant and conflicting gene trees, respectively; Figure S7: Maximum likelihood (ML) phylogenetic tree of “Ardisioids” based on nuclear ribosomal DNA (nrDNA) data. The tree was constructed using RAxML based on nrDNA sequences. Values at the nodes represent bootstrap support from 500 replicates; Figure S8: Species tree of “Ardisioids” based on genome-wide SNPs using WASTER. This comprehensive tree represents the consolidated phylogenetic framework derived from whole-genome variants. Numbers at the branches indicate support values (local bootstrap support), showing the robust relationships (>95.0) between different groups; Table S1: The information of species and molecular data used in this study; Table S2: The newly Genome skimming sequencing data and the assembly information of plastomes in this study. Table S3: Functional annotation, sequence information, and taxon coverage of genes in the plastid gene supermatrix. Table S4: The calibrations imposed by divergence time estimation in MCMCTREE; Table S5: Analysis of Robinson–Foulds (RF), normalized RF distance and weighted RF distances for the plastid dataset under various modeling strategies; Table S6: Model comparison between HiSSE models. Marginal log-likelihood (marginal lnL) was calculated through the stepping-stone sampling algorithm in RevBayes. References [82,83,84,85,86,87] are included in the Supplementary Materials.
