# Supplementary material for: The Evolutionary Significance of Leaf Nodulation: Evidence from Ardisia and Its Relatives (Primulaceae: Myrsinoideae)
Source: Biology (Basel). 2026 Aug 24;15(17):1451. doi: 10.3390/biology15171451 (PMC13564548; doi:10.3390/biology15171451)

Figure S1: Maximum likelihood (ML) tree of "Ardisioids" based on unpartitioned plastid genes. The phylogeny was inferred using RAxML based on the concatenated 80 plastid genes alignment matrix under an unpartitioned model. Numbers at the nodes represent bootstrap support values derived from 500 replicates.

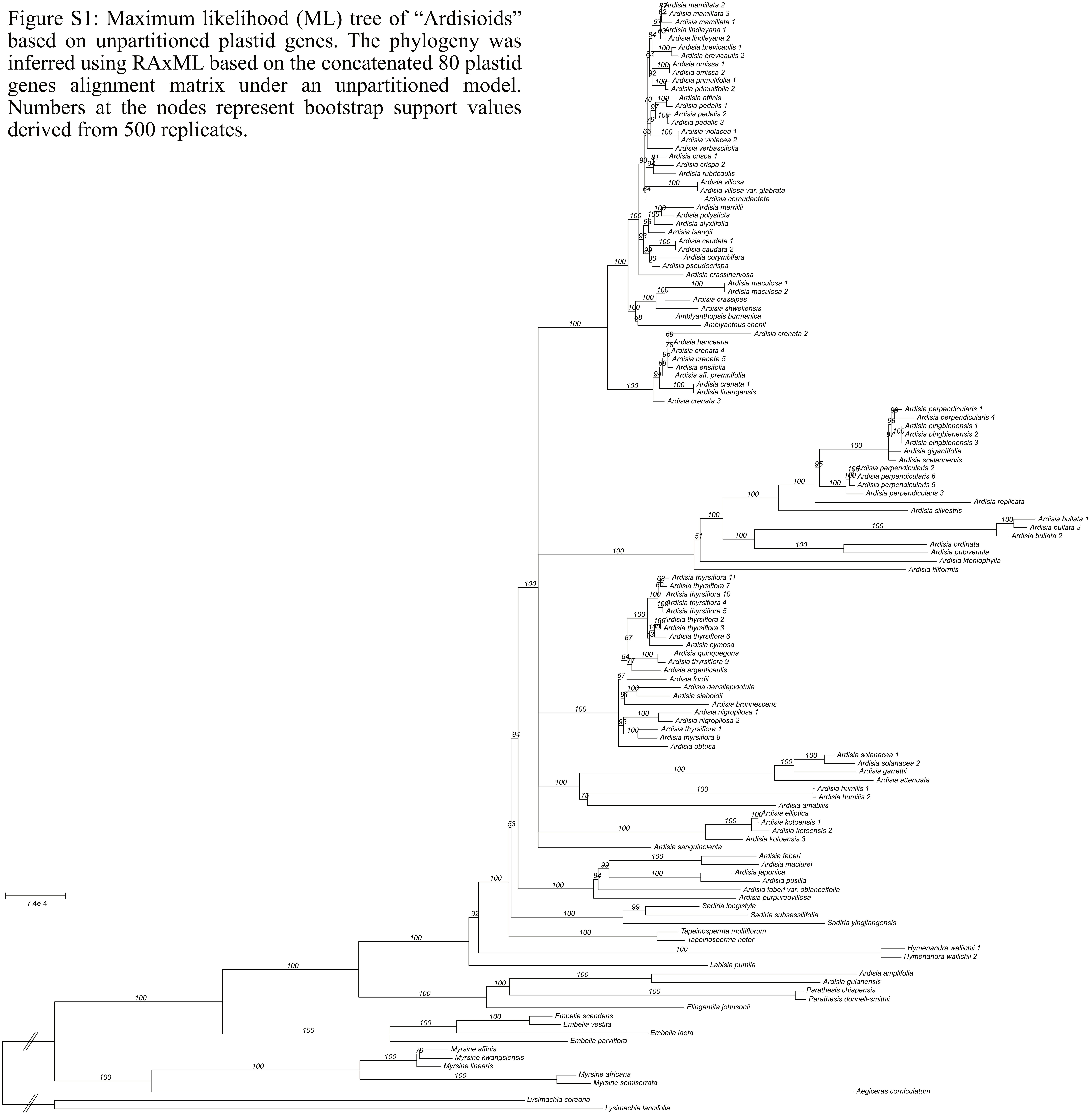

Supplement: Supplementary file 1 [file biology-15-01451-s001.zip › Figure S1 RAxML_CP_nopart.pdf]
