# Supplementary material for: The Evolutionary Significance of Leaf Nodulation: Evidence from Ardisia and Its Relatives (Primulaceae: Myrsinoideae)
Source: Biology (Basel). 2026 Aug 24;15(17):1451. doi: 10.3390/biology15171451 (PMC13564548; doi:10.3390/biology15171451)

Figure S2: Maximum likelihood (ML) tree of "Ardisioids" based on plastid data partitioned by codon position. The phylogeny was inferred using RAxML with the dataset partitioned according to the codon positions (1st, 2nd, and 3rd) of the protein-coding genes. Numbers at the nodes represent bootstrap support (BS) values derived from 500 replicates.

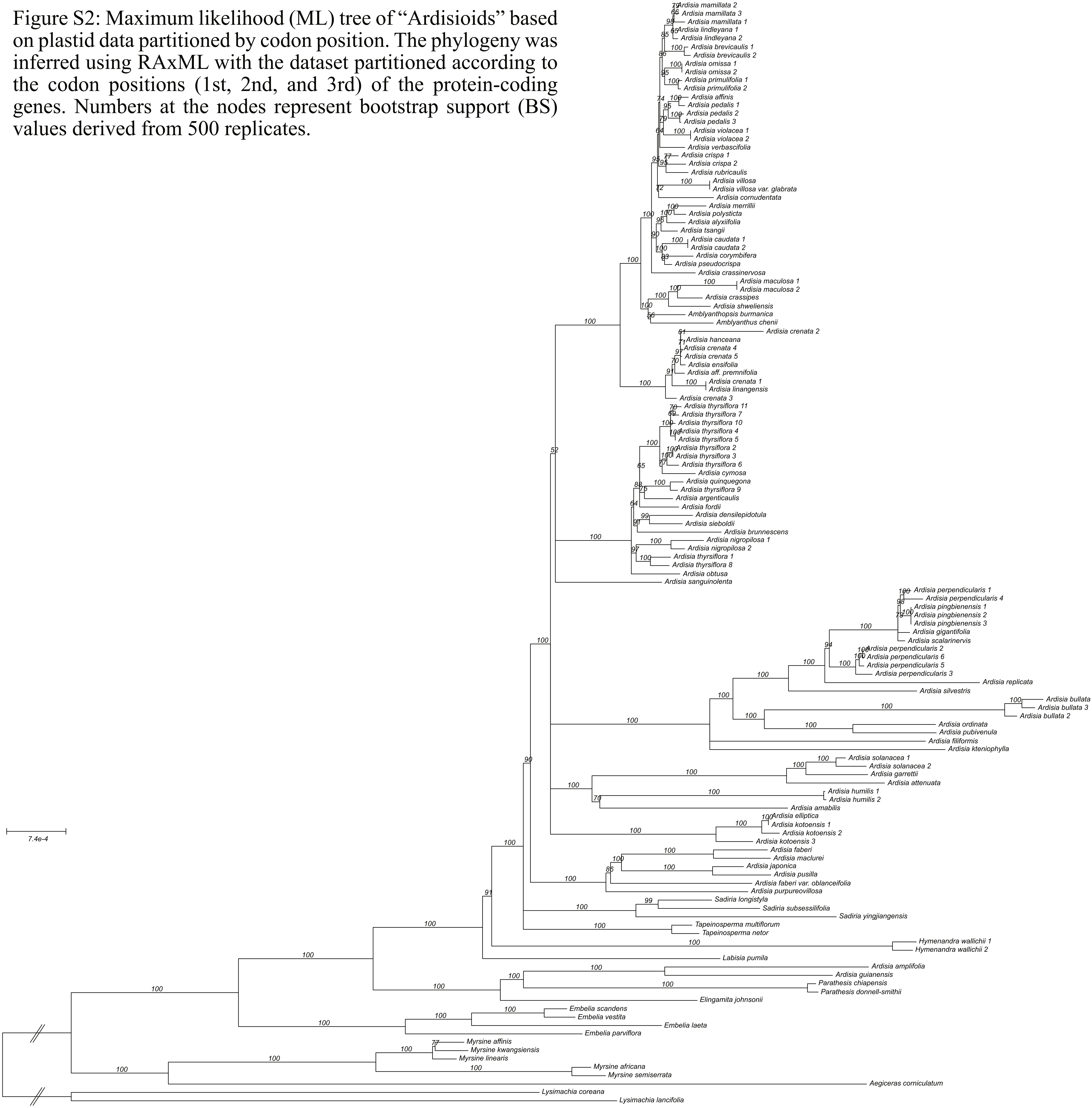

Supplement: Supplementary file 1 [file biology-15-01451-s001.zip › Figure S2 RAxML_CP_partcodon.pdf]
