# Supplementary material for: The Evolutionary Significance of Leaf Nodulation: Evidence from Ardisia and Its Relatives (Primulaceae: Myrsinoideae)
Source: Biology (Basel). 2026 Aug 24;15(17):1451. doi: 10.3390/biology15171451 (PMC13564548; doi:10.3390/biology15171451)

Figure S3: Maximum likelihood (ML) tree of "Ardisioids" based on plastid data partitioned by gene. The phylogeny was inferred using RAxML with a gene-based partitioning scheme, where each gene was treated as an independent partition. Numbers at the nodes represent bootstrap support values derived from 500 replicates.

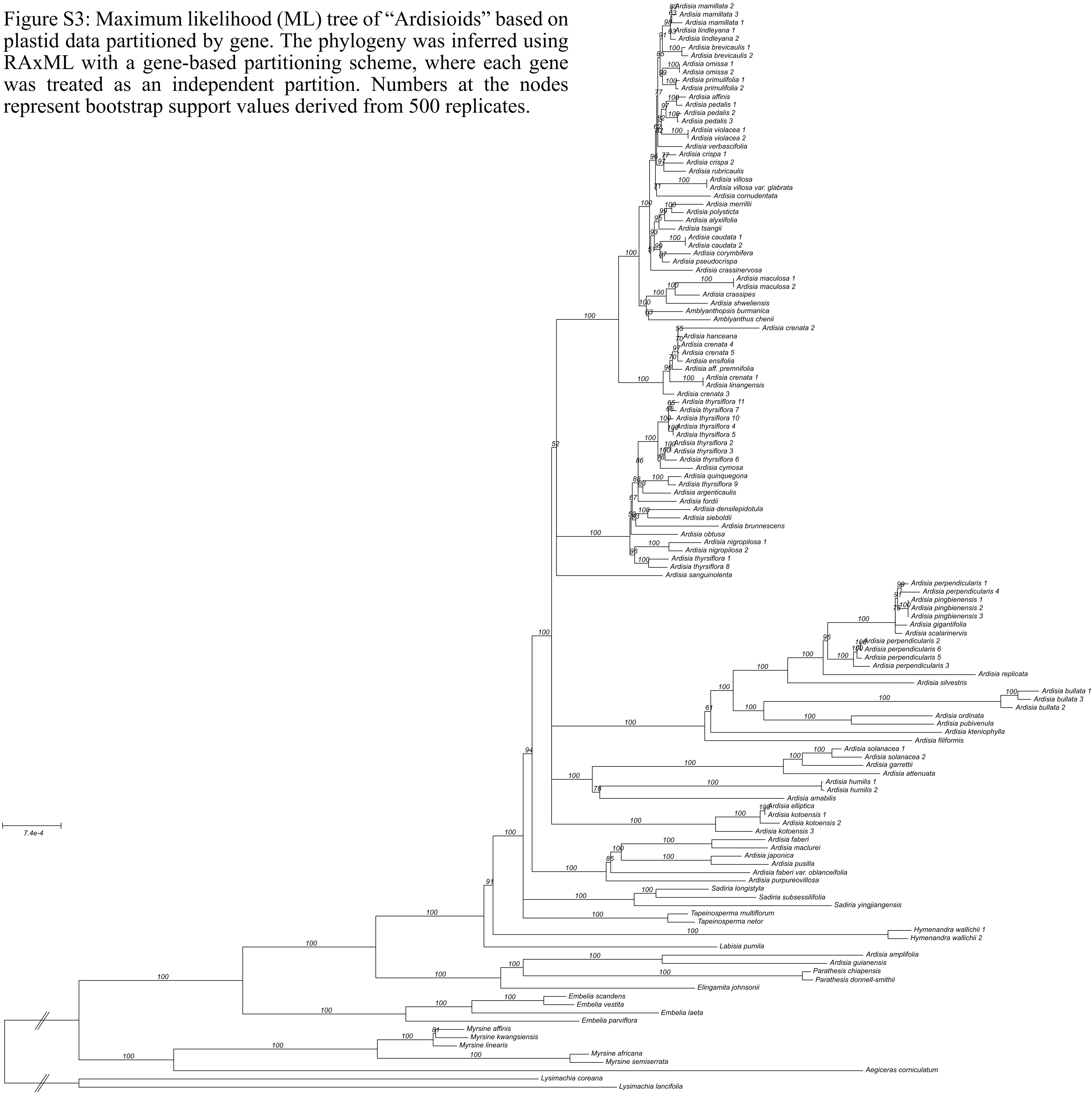

Supplement: Supplementary file 1 [file biology-15-01451-s001.zip › Figure S3 RAxML_CP_partgene.pdf]
