# Supplementary material for: The Evolutionary Significance of Leaf Nodulation: Evidence from Ardisia and Its Relatives (Primulaceae: Myrsinoideae)
Source: Biology (Basel). 2026 Aug 24;15(17):1451. doi: 10.3390/biology15171451 (PMC13564548; doi:10.3390/biology15171451)

Figure S5: Maximum likelihood (ML) tree of "Ardisioids" based on plastid data partitioned by gene using IQ-TREE. The phylogeny was inferred using IQ-TREE with a gene-based partitioning scheme. The numbers above and below the nodes represent ultrafast bootstrap (UFB) supports and SH-aLRT values derived from 1000 replicates.

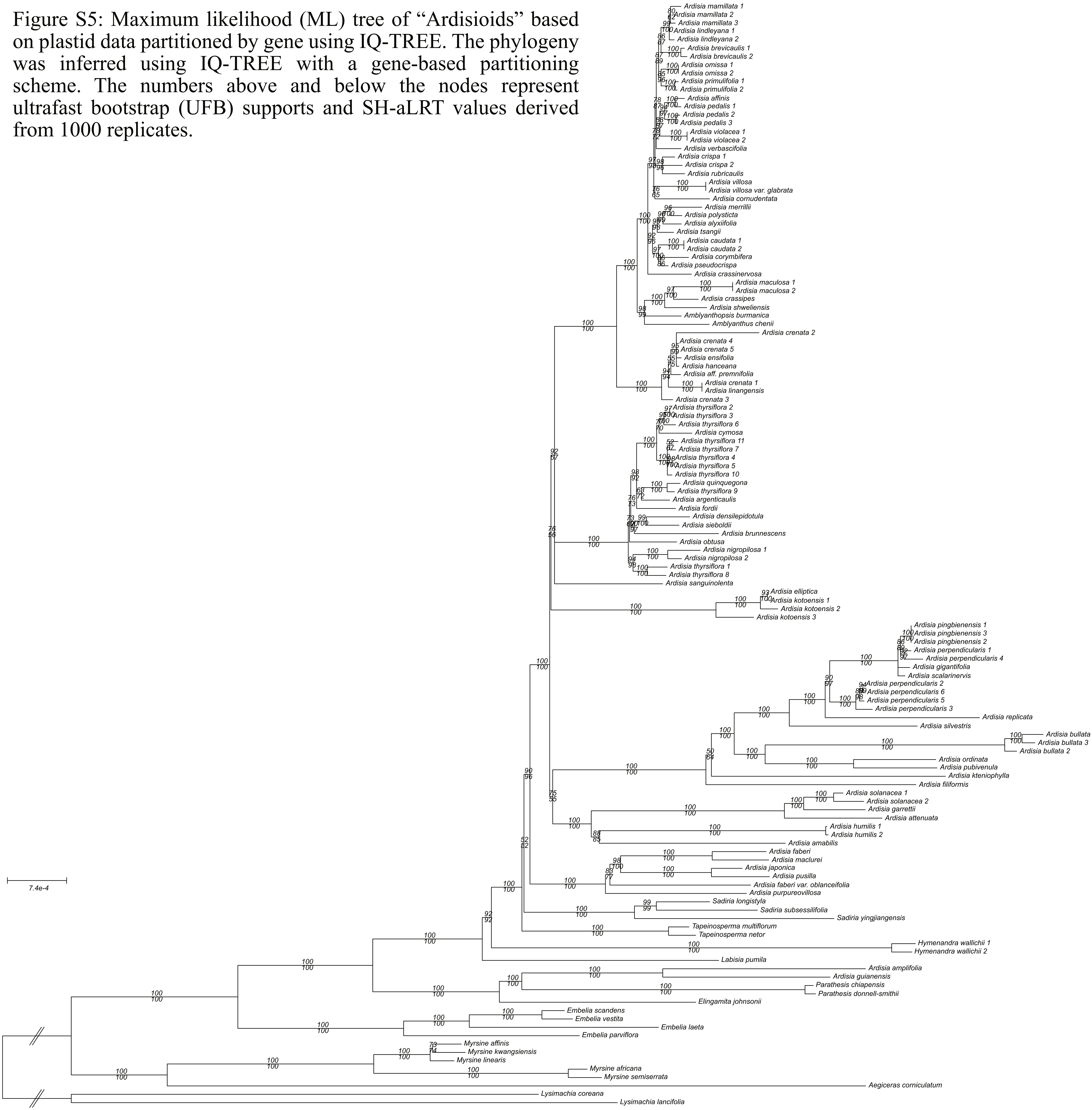

Supplement: Supplementary file 1 [file biology-15-01451-s001.zip › Figure S5 IQTree partgene.pdf]
