# Supplementary material for: The Evolutionary Significance of Leaf Nodulation: Evidence from Ardisia and Its Relatives (Primulaceae: Myrsinoideae)
Source: Biology (Basel). 2026 Aug 24;15(17):1451. doi: 10.3390/biology15171451 (PMC13564548; doi:10.3390/biology15171451)
Supplement: Supplementary file 1 [file biology-15-01451-s001.zip › Figure S6 phyparts_pies.pdf]

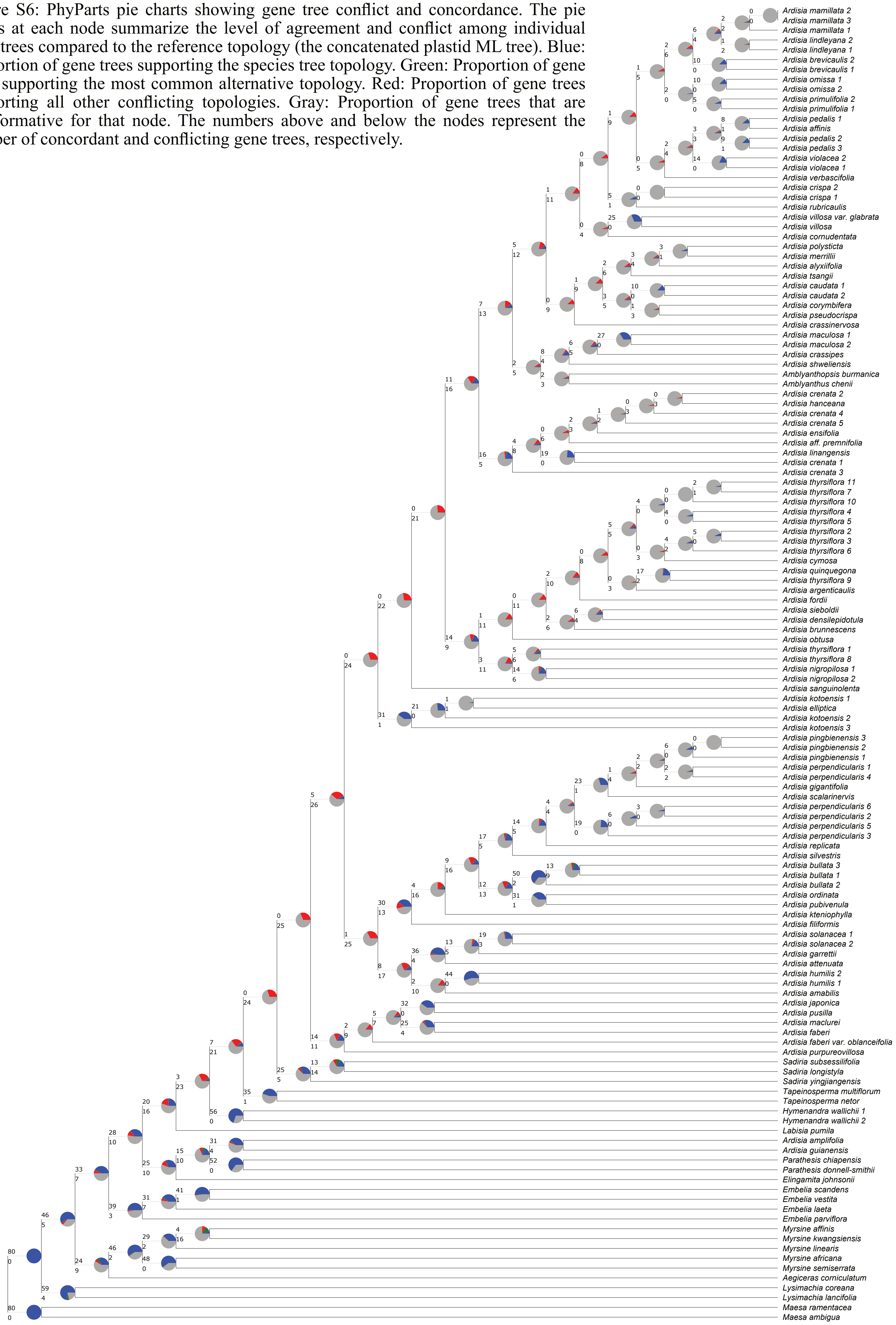

Figure S6: PhyParts pie charts showing gene tree conflict and concordance. The pie charts at each node summarize the level of agreement and conflict among individual gene trees compared to the reference topology (the concatenated plastid ML tree). Blue: Proportion of gene trees supporting the species tree topology. Green: Proportion of gene trees supporting the most common alternative topology. Red: Proportion of gene trees supporting all other conflicting topologies. Gray: Proportion of gene trees that are uninformative for that node. The numbers above and below the nodes represent the number of concordant and conflicting gene trees, respectively.
