# Supplementary material for: The Evolutionary Significance of Leaf Nodulation: Evidence from Ardisia and Its Relatives (Primulaceae: Myrsinoideae)
Source: Biology (Basel). 2026 Aug 24;15(17):1451. doi: 10.3390/biology15171451 (PMC13564548; doi:10.3390/biology15171451)

Figure S7: Maximum likelihood (ML) phylogenetic tree of "Ardisioids" based on nuclear ribosomal DNA (nrDNA) data. The tree was constructed using RAxML based on nrDNA sequences. Values at the nodes represent bootstrap support from 500 replicates.

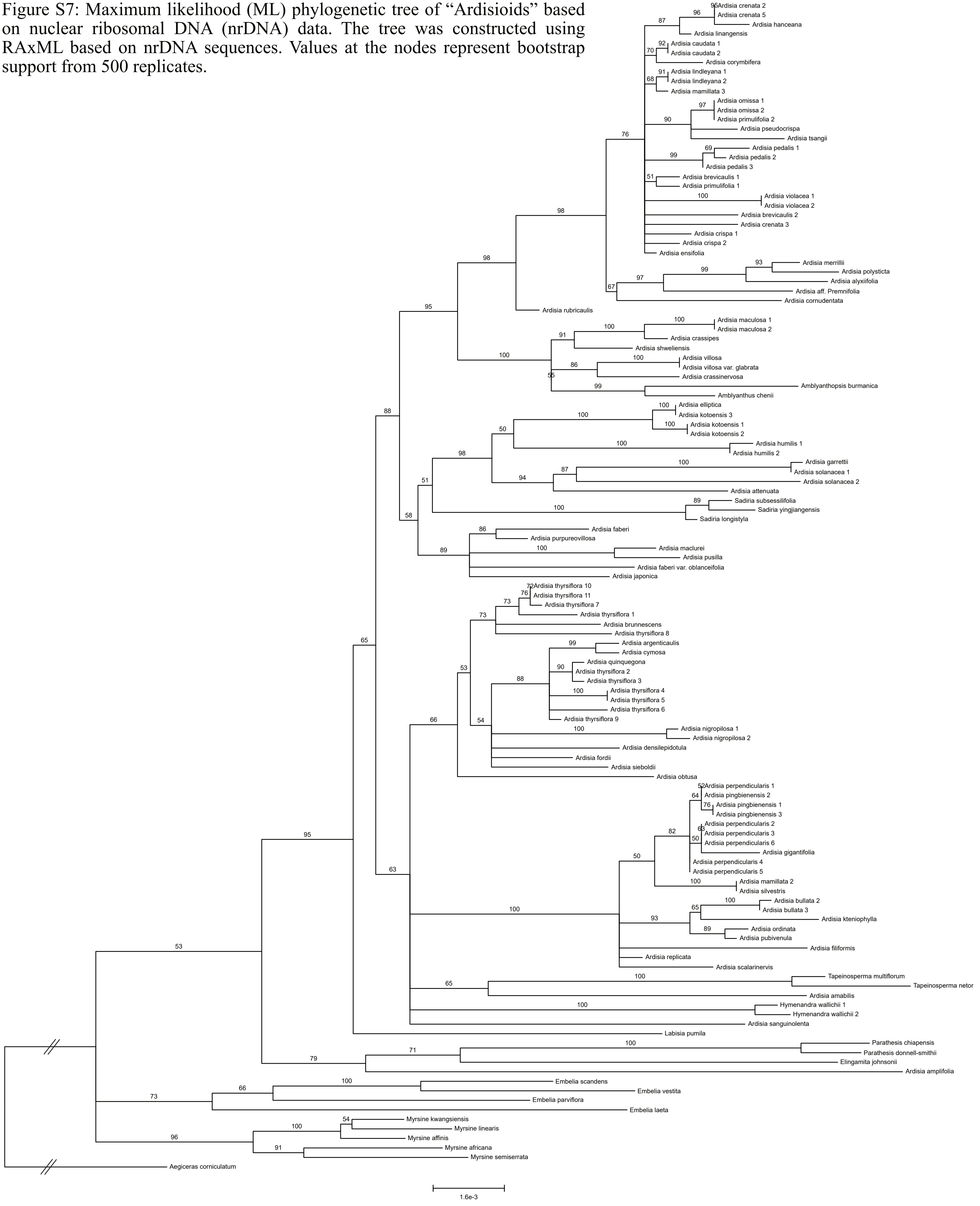

Supplement: Supplementary file 1 [file biology-15-01451-s001.zip › Figure S7 RAxML nrDNA.pdf]
