# Supplementary material for: The Evolutionary Significance of Leaf Nodulation: Evidence from Ardisia and Its Relatives (Primulaceae: Myrsinoideae)
Source: Biology (Basel). 2026 Aug 24;15(17):1451. doi: 10.3390/biology15171451 (PMC13564548; doi:10.3390/biology15171451)

Figure S8: Species tree of “Ardisioids” based on genome-wide SNPs using WASTER. This comprehensive tree represents the consolidated phylogenetic framework derived from whole-genome variants. Numbers at the branches indicate support values (local bootstrap support), showing the robust relationships (>95.0) between different groups

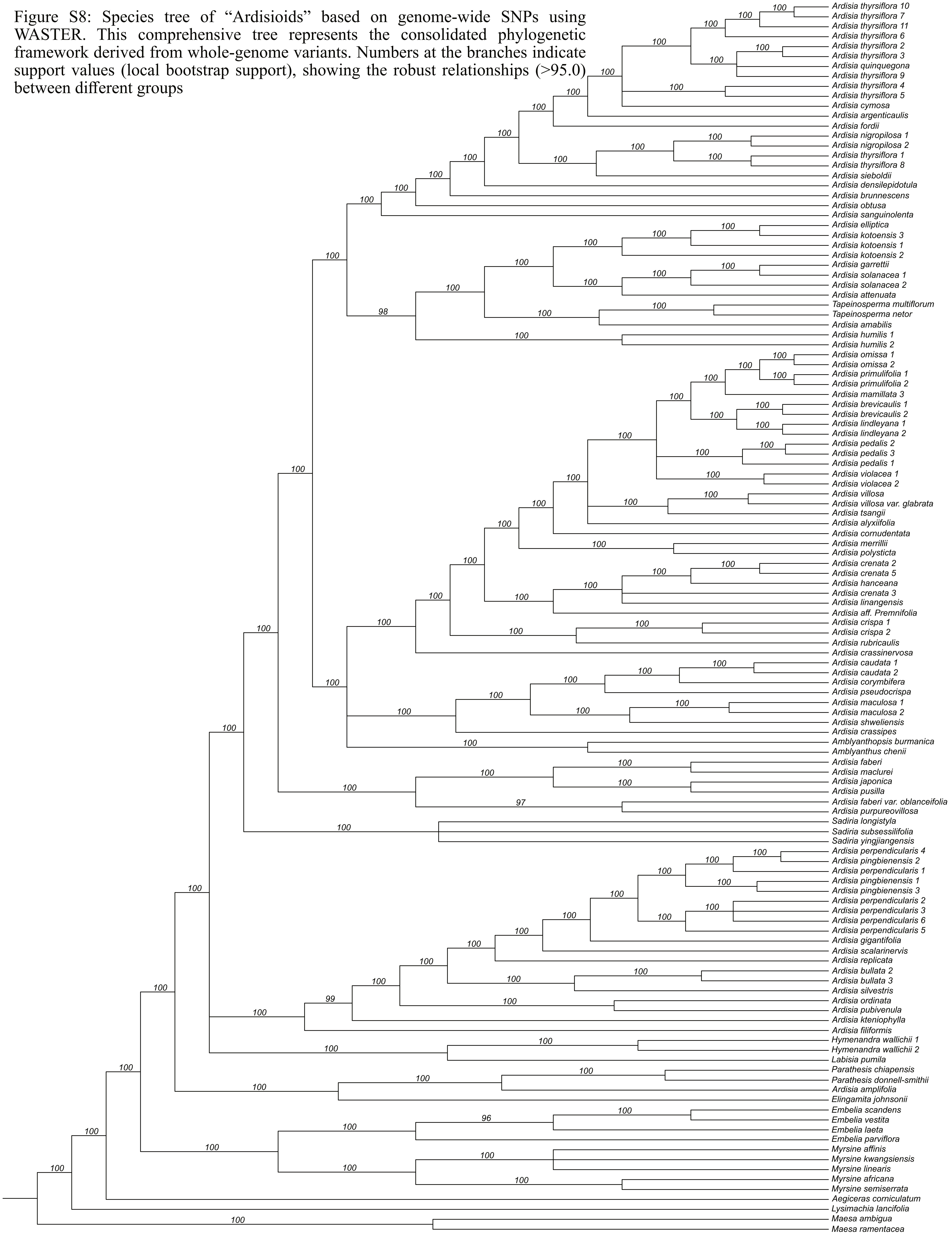

Supplement: Supplementary file 1 [file biology-15-01451-s001.zip › Figure S8 Waster Tree.pdf]
